# Antisaccade performance in schizophrenia: a neural model of decision making in the superior colliculus

**DOI:** 10.3389/fnins.2014.00013

**Published:** 2014-02-11

**Authors:** Vassilis Cutsuridis, Veena Kumari, Ulrich Ettinger

**Affiliations:** ^1^Institute of Molecular Biology and Biotechnology, Foundation for the Research and Technology-Hellas (FORTH)Heraklion, Greece; ^2^Department of Psychology, Institute of Psychiatry, King's College LondonLondon, UK; ^3^South London and Maudsley NHS Foundation Trust, NIHR Biomedical Research Centre for Mental HealthLondon, UK; ^4^Department of Psychology, University of BonnBonn, Germany

**Keywords:** antisaccade performance, rise-to-threshold model, neural model, superior colliculus, eye movements, schizophrenia

## Abstract

Antisaccade performance deficits in schizophrenia are generally interpreted as an impaired top–down inhibitory signal failing to suppress the erroneous response. We recorded the antisaccade performance (error rates and latencies) of healthy and schizophrenia subjects performing the mirror antisaccade task. A neural rise-to-threshold model of antisaccade performance was developed to uncover the biophysical mechanisms giving rise to the observed deficits in schizophrenia. Schizophrenia patients displayed greater variability in the antisaccade and corrected antisaccade latency distributions, increased error rates and decreased corrected errors, relative to healthy participants. Our model showed that (1) increased variability is due to a more noisy accumulation of information by schizophrenia patients, but their confidence level required before making a decision is unaffected, and (2) competition between the correct and erroneous decision processes, and not a third top-down inhibitory signal suppressing the erroneous response, accounts for the antisaccade performance of healthy and schizophrenia subjects. Local competition further ensured that a correct antisaccade is never followed by an error prosaccade.

## Introduction

Decision making is an accumulation process of evidence about the state of the world and the utility of possible outcomes. A fruitful experimental approach employed by behavioral neuroscientists to understanding how humans and animals make decisions is the antisaccade paradigm (Hallett, 1978). In the antisaccade paradigm subjects are required to suppress an erroneous saccade (error prosaccade) toward a peripheral stimulus and instead make an eye movement to an equidistant position in the opposite hemifield (mirror antisaccade). The paradigm requires at least two decision processes: (1) suppression (or inhibition) of an error prosaccade toward the peripheral stimulus, and (2) generation of a volitional saccade to the mirror position (antisaccade) (Everling and Fischer, 1998; Broerse et al., 2001; Munoz and Everling, 2004). Subjects make errors in this paradigm when they look toward the peripheral stimulus instead of performing the antisaccade. The error rate is the most reliable measure of antisaccade performance (Ettinger et al., 2003). Healthy participants typically fail to suppress erroneous prosaccades toward the target on about 20–25% of trials, before correctly saccading toward the mirror image location (Fischer and Weber, 1992; Everling and Fischer, 1998; Smyrnis et al., 2002; Ettinger et al., 2003; Tatler and Hutton, 2007).

In addition to error rates, the antisaccade task yields several other measures that provide important insights into the integrity of the cognitive and neural mechanisms involved in decision making. These include the reaction times (RT) of antisaccades and error prosaccades, the time taken to correct errors (the time between an error prosaccade and subsequent corrective antisaccade), the percentage of errors that are corrected, and various spatial accuracy measures including the amplitude of antisaccades and error prosaccades and the final eye position of correct responses (Hutton and Ettinger, 2006). A large study of young males has reported that error prosaccade and antisaccade RTs are variable within subjects and across subjects (Evdokimidis et al., 2002; Smyrnis et al., 2002). In that study, the mean prosaccade RT was reported to be 208 ms (*SD* = 38), whereas the mean antisaccade RT was 270 ms (*SD* = 39 ms) (Evdokimidis et al., 2002). The corrective antisaccade RT was 146 ms (*SD* = 55 ms) (Evdokimidis et al., 2002). The error rate was reported to be 23% (Evdokimidis et al., 2002; Smyrnis et al., 2002).

Although healthy participants typically make few errors (Everling and Fischer, 1998), patients with frontal lobe lesions (Guitton et al., 1985) and patients suffering from schizophrenia (Fukushima et al., 1988) make more antisaccade errors and their antisaccade RTs are more variable within and across subjects (Fukushima et al., 1988; Hutton et al., 1998; Karoumi et al., 1998; Brownstein et al., 2003). The antisaccade performance deficit is usually reported to be a deficit in top–down inhibition control of the erroneous response or a deficit in response generation of the antisaccade (Everling and Fischer, 1998; Broerse et al., 2001; Curtis et al., 2001; Brownstein et al., 2003). In these accounts, the inhibition process is thought to be independent from the volitional generation of the antisaccade.

Recent experiments of antisaccade performance have emphasized the parallel nature of saccade programming in the antisaccade task (Massen, 2004; Munoz and Everling, 2004; Reuter and Kathmann, 2004). They argue that after a peripheral stimulus is presented a competition begins between the exogenously triggered prosaccade and the endogenously initiated antisaccade. Massen (2004) argued that if the volitional antisaccade is programmed fast enough (e.g., reaches some threshold for activation), then it will win the competition, and the reflexive-like saccade will be cancelled. Alternatively, if the reflexive-like prosaccade is programmed fast enough (or the computation for the antisaccade is too slow) an erroneous prosaccade will be made first, and the correct antisaccade will follow. Munoz and Everling (2004) instead argued that “errors occur when processes related to the initiation of the prosaccade toward the target are inadequately “handicapped,” resulting in an increased likelihood of it reaching the threshold for saccade triggering.” This account favors the concept of an active inhibitory mechanism as being critical to antisaccade performance.

Over the years a number of mathematical models of decision making have been advanced (Carpenter and Williams, 1995; Ratcliff et al., 1999; Carpenter, 2000; Reddi and Carpenter, 2000; Usher and McClelland, 2001; Cutsuridis et al., 2007a; Noorani and Carpenter, 2013). In these models the process of decision making often involves a gradual accumulation of information concerning the various potential responses. When the target appears a decision process starting at some baseline level *S*_0_, which represents the prior expectation, begins to rise at a constant rate *r* until it reaches a threshold *S*_*T*_, which represents the confidence level required before the commitment to a particular course of action. Once the decision signal crosses *S*_*T*_, then a response toward the target is initiated. Response time (RT) is then the time from the onset of the decision process till when the decision signal crosses *S*_*T*_. The rate of rise is sometimes assumed to vary randomly from trial to trial, with a mean μ and variance σ^2^ (Reddi and Carpenter, 2000). Changes in the baseline level of activity, the rate of rise or the threshold often result in changes in response latency. Prior expectation and level of activation of intention influence the baseline levels of activation. Carpenter in his LATER model (1981) proposed if the cumulative RT distribution is plotted against 1/RT on reciprobit scale, then the resulting straight line can be used as a diagnostic tool to assess the contribution of different factors influencing the experimental results. In a choice reaction time task, the various choices are represented by different straight lines. If, for example, the lines swiveled by the threshold *S*_*T*_ (Reddi and Carpenter, 2000), then the mean and variances of the lines were unequal. If the lines were shifted by μ, then the slopes (1/σ) of the lines were equal, but their latency medians were not (Reddi et al., 2003). If the lines crossed, then the slopes were not equal, but their medians were (Nakahara et al., 2006).

Other decision making models extending the notion of decision making as a gradually accumulating process addressed the question of whether a third signal, inhibitory in nature, is needed to prevent the unwanted decision from being expressed after the correct decision is expressed first. A recent study (Noorani and Carpenter, 2013) investigating the antisaccade performance of normal participants has suggested that such a third STOP process is necessary to suppress the error prosaccade that would otherwise be generated. A recent neural network implementation of antisaccade performance of healthy subjects (Cutsuridis et al., 2007a) have instead proposed that competition between the volitional antisaccade and erroneous prosaccade and not inhibition of the erroneous prosaccade by a third signal is sufficient to accurately reproduce the error rate and correct antisaccade and error prosaccade latency distributions of antisaccade data from a large cohort, while at the same time replicating the finding that whilst erroneous prosaccades toward the target are nearly always followed by a correct antisaccade, the opposite never occurs.

Here we extend the Cutsuridis and colleagues model of antisaccade performance (Cutsuridis et al., 2007a) into the realm of schizophrenia. We quantitatively answer why the antisaccade performance of patients with schizophrenia is so poor and whether their poor performance is due to a deficit in the top–down inhibitory control of the erroneous response. Our model successfully reproduced the correct antisaccade, error prosaccade and corrected antisaccade latency distributions (median and variance) as well as the error rates and the percentage of errors corrected of both normal and schizophrenia subjects. Our model showed that the increased variability in the antisaccade and corrected antisaccade RT distributions of schizophrenia suffering subjects are due to a more noisy accumulation of information (μ and σ) and that their prior probability (*S*_0_) and confidence level (decision threshold level *S*_*T*_) required before commitment to a particular action are unaffected by the disease. Our model in line with previous modeling studies (Cutsuridis et al., 2007a) showed that local competition between the erroneous and correct decision signals and not a third top–down inhibitory signal that suppresses the erroneous response can account for the antisaccade performance in both healthy and schizophrenia subjects. Local competition further ensured that a correct antisaccade is never followed by an error prosaccade.

## Materials and methods

### Experimental data

#### Sample description

The antisaccade performance (error rates and latencies) of 45 patients (25 males and 20 females) with a DSM-IV diagnosis of schizophrenia (mean age = 44.69 years; *SD* = 11.62) and 34 healthy controls (15 males and 19 females) (mean age = 34 years, *SD* = 13.40) without DSM-IV diagnosis was recorded. Patients were recruited from outpatient services within and around South London. Healthy controls were recruited from the same geographical area using advertisements. The patients' diagnoses (First et al., 1997) and the absence of diagnoses in the controls were established using the Structured Clinical Interviews (First et al., 1996). All participants were free of neurological conditions, head trauma with loss of consciousness, and drug or alcohol abuse. Patients did not have any additional Axis I disorders and controls did not have any first-degree relatives with psychosis. All participants were right-handed. Patients were treated with typical antipsychotics for at least 6 weeks (chlorpromazine equivalents in mg/day: mean = 199.11, *SD* = 120.62, range = 31.25–550.00).

The gender distribution did not significantly differ between groups (*p* = 0.31). The statistical test used for gender distribution was a chi-square goodness of fit test. However, the two groups differed significantly in age, with the patient group being older than the control group [*t*_(77)_ = 3.79, *p* < 0.001].

All participants provided written informed consent before participation and the study procedures were approved by the local research ethics committee.

#### Eye movement task

Tasks were identical to a previously described protocol (Ettinger et al., 2003). A white target of circular shape (0.3°) was presented on the black background of a 17-inch monitor 57 cm from participants. Head movements were minimized using a chinrest.

#### Antisaccade task

An antisaccade trial began with the target in the central location for a random duration of 1000–2000 ms. The target then stepped to one of four peripheral locations (±6°, ±12°) where it remained for 1000 ms. Each peripheral location was used 15 times in random order. There were four practice trials. Participants were instructed to look at the target while in the central position and then to look to the exact mirror image location of the peripheral target as fast and accurately as possible.

### Eye movement recording and analysis

Left eye movements were recorded using infrared oculography (IRIS 6500 by Skalar Medical BV). Signals were converted from analog to digital by a 4-channel, 12-bit analog-to-digital converter sampled at 500 Hz. Interactive software was used for analysis of eye movement data (inter- and intra-rater reliability: *r* > 0.90). Data were scored blind to group status. Eye-blinks were identified on the basis of position and velocity charts.

#### Antisaccade task

**Figure A1:**
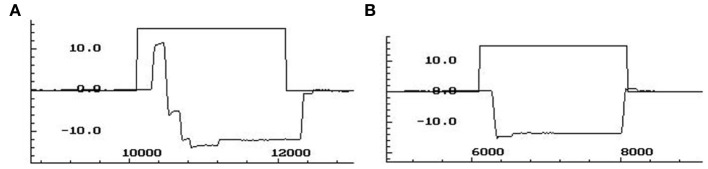
**(A)** A trace of a trajectory of an antisaccade direction error with subsequent corrective saccade. **(B)** A trace of a trajectory of a directionally correct antisaccade. X-axis: time in ms; y-axis: degree of visual angle.

A correct antisaccade trial occurred when a primary saccade was performed in the opposite direction of the peripheral target. An antisaccade error was counted when a primary saccade was performed toward the peripheral target. The antisaccade error rate reflects the percentage of error trials over the total number of valid trials. Antisaccade latency (ms) of correct antisaccades was measured using above criteria. Figure A1 depicts traces of antisaccade and corrected antisaccade trajectories.

### Neural network model

#### Architecture

The model was a competitive neural network of the intermediate layer of the superior colliculus (SC) (Figure 2A). SC has been suggested to play a role in the formation of the final motor command, which then is sent to the eye muscles for an eye movement generation. Model SC neurons were represented as rate nodes. The total number of nodes in the network was N. The left N/2 nodes in the network represented the left SC, whereas the right N/2 nodes represented the right SC. Short-range lateral excitation and long distance lateral inhibition was assumed between all nodes in model. The internal state *x*_*i*_(*t*) of each node (Figure 2C) is governed by

(1) $$\tau \frac{dx_{i}(t)}{dt}=-x_{i}(t)+\sum_{j}w_{ij}A_{j}(t)+I_{\text{ext}}(t)+I_{n}$$

where τ is the integration time constant, *w*_*ij*_ is the synaptic efficacy from node *i* to node *j*, *A*_*j*_ is the activity function of node *j*, *I*_ext_ is the external input reactive (*I*_*r*_) or planned (*I*_*p*_) decision signal that each SC (left or right) received from cortical areas (posterior parietal cortex or frontal cortex), and *I*_*n*_ is the background noise. In the left SC τ takes values from a normal distribution with mean (μ_1_) and standard deviation (σ_1_), whereas in the right SC τ takes values from a different normal distribution with mean (μ_2_) and standard deviation (σ_2_) (see Table 3 for values).

The activity function *A*_*j*_(*t*) of a node *j* representing the average membrane potential is given by a sigmoid function

(2) $$A_{i}(t)=\frac{1}{1+\text{exp}(\text{-}\beta {\text{x}}_{\text{i}}(\text{t}))}-\theta$$

where β is the steepness and θ is the offset of the sigmoid.

The lateral interaction kernel *w*_*ij*_, which allows for lateral interactions between nodes in the same colliculus and between nodes located in opposite colliculi sites is chosen to be a shifted Gaussian, it depends only on the spatial distance between nodes and it is positive for nearby nodes to the node activated by the input and negative for distant nodes (Figure 2B) (Trappenberg, 2009):

(3) $$w_{ij}=B\cdot \left(\frac{1}{\sqrt{4\pi }\sigma }e^{-{\left((i-j)\cdot \Delta x\right)}^{2}/\text{4}\sigma^{2}}-C\right)$$

where *B* and *C* are free parameters and σ is a spatial parameter.

#### Model inputs

The model is activated by two inputs: the reactive input (*I*_*r*_), which represents the error prosaccade and a planned input (*I*_*p*_), which represents the correct antisaccade. The reactive input activates a node and two of each nearest neighbors on each side in the left SC and it is thought to originate from the posterior parietal cortices (Munoz and Everling, 2004). The planned input activates the mirror node and its two nearest neighbor nodes on each side in the right SC and originates from the frontal cortices (Munoz and Everling, 2004). The strengths of the external inputs are not equal (*I*_*p*_ > *I*_*r*_; see Table 3 for values).

The reactive input is presented first at time *t* = 50 ms. The planned input is presented at *T* ms after, where *T* varies from 0, 10, 30, and 50 ms. Becker (1989) reported that the difference in the afferent delays of the reactive and planned decision signals (inputs) is close to 50 ms. Both inputs remain active for 600 ms.

#### Implementation

The whole system of differential and algebraic equations is implemented using MATLAB 2009b (The MathWorks, Inc, Natick, MA). Differential equations are integrated numerically using MATLAB's ordinary differential equation solver, *ode45*. The relative (error) tolerance is set to 10^−4^. Simulations that demonstrate how to replicate all the reported effects can be obtained by directly emailing the corresponding author at vcutsuridis@gmail.com.

### Data analysis

Saccade reaction time (RT) is defined as the time interval from the onset of peripheral stimulus till the time of the first detectable eye movement. In the model, saccade RT is estimated as the time interval from the onset of the reactive input till the time the activity of the model neurons reached the threshold (parameter *T*_*h*_ in Table 3), thus an eye movement command is generated, plus an additional 30 ms (approximated time required for the neuronal signal to reach the eye muscles) (Sparks, 1978).

The experimental and simulated control and patient RTs are divided into three behavioral categories: (1) error prosaccades, (2) antisaccades, and (3) corrected antisaccades (the time between an error prosaccade and the subsequent corrected antisaccade).

For our data and statistical analysis we replicate the measures reported in Smyrnis et al. (2009). The experimental median RT and the coefficient of variation of RT for the three behavioral categories (error prosaccade, antisaccade and corrected antisaccade) are calculated for each individual (34 controls and 45 patients). Similarly, the model median RT and coefficient of variation of RT for the three categories (error prosaccade, antisaccade and corrected antisaccade) are calculated for each simulated subject (control subject vs. patient subject) from the 5000 model trials. The coefficient of variation for both experimental and model RT is defined as the inter-quartile RT range (Q_75_–Q_25_) divided by the median RT (Smyrnis et al., 2009). Group means of experimental and model controls and patients for these measures are compared using a *t*-test with different variance estimates.

We estimate the average cumulative distribution for each category (error prosaccade, antisaccade and corrected antisaccade) by organizing the RTs for each subject in ascending order and calculating the percentile values in increments of 5% (the RT at 5, 10, 15, 20, …, 95, 100%). The percentile values are then averaged across each group to give the average group percentile values for error prosaccades, antisaccades and corrected antisaccades, which are then plotted in the average cumulative distribution (controls vs. patients). Ratcliff (1977) showed that the average distribution retains the basic shape characteristics of the individual distributions. In order to test the difference between the group distributions for patients and controls, we use the Wilcoxon signed rank test (*signrank* function in MATLAB).

Carpenter and Williams (1995) showed that if the cumulative RT distribution is plotted using 1/RT in a reciprobit plot, then the RTs will fall on a straight line. Thus, we transformed the average cumulative distribution data of RT (error prosaccade, antisaccade and corrected antisaccade) for the experimental and simulated controls and patients in a reciprobit plot and computed the best-fitting regression line for each group using the regression coefficients that our model produced. An R correlation coefficient was estimated to assess how good fit was the modeled regression line to the experimental data. We then compared the two simulated regression lines for the patient and control groups using the homogeneity of slopes and intercepts regression analysis described in Wuensch (2007).

## Results

### Experimental latency distributions

**Figure 1:**
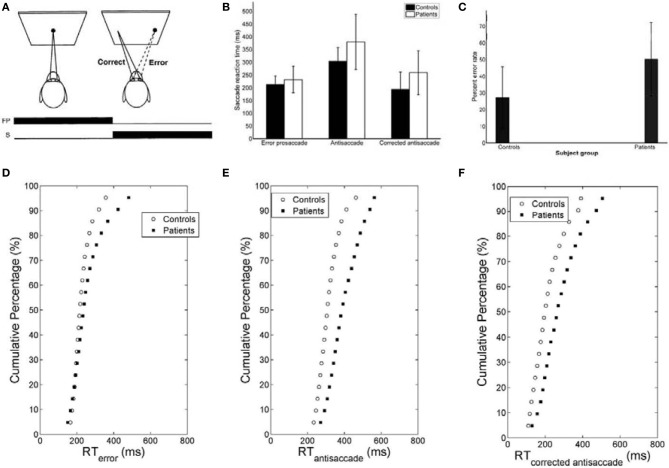
**(A)** Mirror antisaccade task. A participant fixates on a stimulus (FP) centered on the screen. Once a peripheral stimulus (S) appears the participant must suppress the saccade toward the peripheral stimulus (error prosaccade) and instead make an eye movement to equidistant position in the opposite hemifield (antisaccade). **(B)** Mean of median error prosaccade, antisaccade, and corrected antisaccade reaction times (RTs) for controls and patients. **(C)** Mean percent error rate of controls and patients performing the mirror antisaccade task. **(D–F)** Average cumulative percent distributions of error prosaccades **(D)**, antisaccades **(E)**, and corrected antisaccades **(F)** for controls and patients.

The mirror antisaccade task (Figure 1A) was identical to a previously described protocol (Ettinger et al., 2003). The mean inter-individual of the median intra-individual RT for the error prosaccades was found to be 213.26 ms (*SD* = 33.52) for the controls and 232.29 ms (*SD* = 51.61) for the patients (Figure 1B; see Table 1). This 19.03 ms difference was not statistically significant [*t*_(77)_ = 1.87, *P* = 0.06]. The RT distributions for patients were in many cases much broader than those for the controls, indicating a larger RT variability. The group coefficient of variation of RT was not significantly larger for the controls (0.24, *SD* = 0.1) than for the patients (0.21, *SD* = 0.23) [*t*_(111)_ = 0.51, *P* = 0.61] (see Table 2).

**Table 1:** **Simulated median saccade reaction times and their standard deviations and percent error rates for controls and patients with schizophrenia**.

	**Median RT in ms**			**% Error rate**
	**Error prosaccade**	**Antisaccade**	**Corrected antisaccade**	
Controls	212.85	308.10	181.17	15.72 (**27.07**)
	(**213.26**, ***SD*** = **33.52**)	(**304.09, ***SD*** = **52.56****)	(**193.76**, ***SD*** = **66.78**)	
Patients	230.32	372.33	250.07	40.12 (**50.26**)
	(**232.29**, ***SD*** = **51.61**)	(**379.98**, ***SD*** = **108.22**)	(**258.82**, ***SD*** = **86.07**)	

Bold values in parentheses correspond to experimentally estimated means of medians of saccade RTs and their standard deviations and percent error rates for controls and patients.

**Table 2:** **Simulated coefficients of variation (CV) of error prosaccades, antisaccades and corrected antisaccades for controls and patients with schizophrenia performing the mirror antisaccade task**.

	**Coefficient of variation (CV)**		
	**Error prosaccade**	**Antisaccade**	**Corrected antisaccade**
Controls	0.21	0.17	0.44
	(**0.24**, ***SD*** = **0.10**)	(**0.24**, ***SD*** = **0.07**)	(**0.56**, ***SD*** = **0.22**)
Patients	0.36	0.28	0.5
	(**0.21**, ***SD*** = **0.23**)	(**0.18**, ***SD*** = **0.2**)	(**0.3**, ***SD*** = **0.32**)

Bold values in parenthesis correspond to experimentally estimated ones including their standard deviations.

The greater variability of error prosaccade RTs for patients implied a shape difference in the RT distribution for this group. An average cumulative RT distribution for each group (controls vs. patients) (Figure 1D) was computed by organizing the RT for each subject in ascending order and percentile values were calculated (e.g., the RT for the 5% percentile, the 10% percentile, the 15% percentile, …, the 95% percentile, the 100% percentile). The percentile values were then averaged across the group to give average group percentile values. To test the difference between the group distributions for patients and controls, a Wilcoxon signed rank test was used. It can be observed that the two cumulative distributions differ in shape and this difference was significant (*Z* = 3.173, *P* = 0.001).

A similar analysis was used for the antisaccades and corrected antisaccades for both the controls and patients. The mean inter-individual of the median intra-individual RT for the antisaccades was 304.09 ms (*SD* = 52.56) for the controls and 379.98 ms (*SD* = 108.22) for the patients (Figure 1B; see Table 1). This 75.89 ms difference was statistically significant [*t*_(77)_ = 3.76, *P* < 10^−3^]. The coefficient of variation of RT was not significantly larger for the controls (0.24, *SD* = 0.07) than for the patients (0.18, *SD* = 0.2) [*t*_(111)_ = 1.69, *P* = 0.09] (see Table 2).

The average cumulative RT distribution for each group (controls vs. patients) (Figure 1E) was computed as before. To test the difference between the group distributions for patients and controls, a Wilcoxon signed rank test was used. It can be observed that the two cumulative distributions differ in shape and this difference was significant (*Z* = 3.92, *P* < 10^−4^).

The mean inter-individual of the median intra-individual RT for the corrected antisaccades was 193.76 ms (*SD* = 66.78) for the controls and 258.82 ms (*SD* = 86.07) for the patients (Figure 1B; see Table 1). This 60.75 ms difference was statistically significant [*t*_(76)_ = 3.64, *P* < 10^−3^]. The coefficient of variation of RT was significantly larger for the controls (0.56, *SD* = 0.22) than for the patients (0.30, *SD* = 0.32) [*t*_(111)_ = 4.21, *P* < 10^−3^] (see Table 2).

The average cumulative RT distribution for each group (controls vs. patients) (Figure 1F) was similarly computed and a Wilcoxon signed rank test was used to test the difference between the group distributions for patients and controls. The two cumulative distributions differed in shape and this difference was significant (*Z* = 3.92, *P* < 10^−3^).

### Neural rise-to-threshold model and simulated latencies

**Figure 2:**
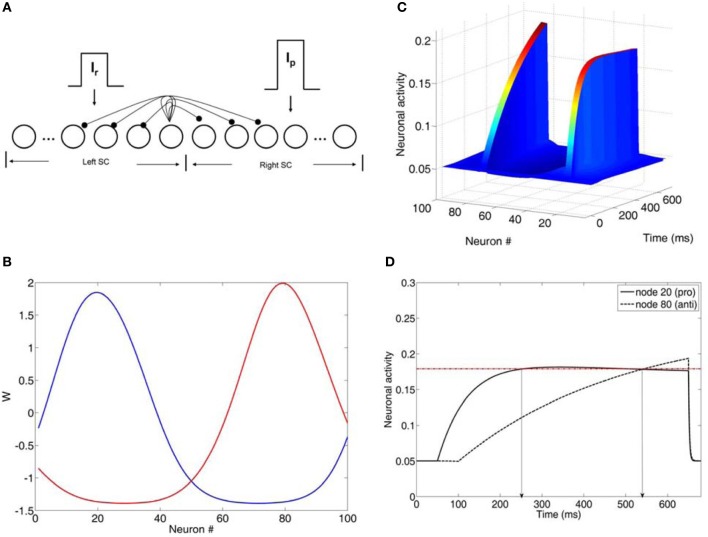
**(A)** Neural network model of the intermediate layer of the superior colliculus. Neurons are represented as nodes. Short-range lateral excitation and long distance lateral inhibition was assumed between all nodes in the network. The left half of the network represented the left SC, whereas the right half represented the right SC. The left SC was activated by a reactive input *I*_*r*_, whereas the right SC was activated by a planned input *I*_*p*_. The strengths of the inputs were not equal (*I*_*p*_ = 1.5^*^I_*r*_). **(B)** Lateral interaction kernels W for nodes 20 and 80 modeled as a shifted Gaussians. The kernels for nodes 20 and 80 were excitatory for the nearby nodes and inhibitory for the distant ones. **(C)** Neuronal activities of all nodes in the network as a function of time (ms). **(D)** Neuronal activity of nodes 20 and 80 as a function of time. Node 20 encoded the reactive input (error prosaccade) and node 80 encoded the planned input (antisaccade). When both activities crossed the threshold (dotted horizontal line), then an eye movement decision was made. In this case, an error prosaccade followed by a corrected antisaccade.

To fit the experimental data, we employed the Cutsuridis and colleagues model of antisaccade performance (Cutsuridis et al., 2007a) and extended it into the realm of schizophrenia (Figure 2A). The model is a neural network implementation of a rise-to-threshold model in the SC tailored to the needs of the antisaccade paradigm. As in the Cutsuridis et al. model (2007a), the decision signals for the volitional antisaccade and reactive prosaccade are integrated in a competitive manner in the intermediate layer of the SC. The neural model had 100 nodes. A node in the left SC (node 20) and its four nearest neighbors (nodes 18, 19, 21, 22) were selected to encode the reactive input (*I*_*r*_) and compute the error prosaccade, and a node in the right SC (node 80) and its four nearest neighbors (nodes 78, 79, 81, 82) were selected to encode the planned input (*I*_*p*_) and compute the antisaccade. The strengths of the inputs were not equal (e.g., *I*_*p*_ = 1.5^*^I_*r*_). In each trial run the reactive input was presented first at time *t* = 50 ms. The planned input was presented at time *t* = *T* ms, where *T* was 50 ms unless mentioned otherwise. Both inputs remained active for 600 ms. The interaction weight matrix between the nodes was chosen to be a shifted Gaussian, which depended on the spatial distance between nodes and it was positive for nearby nodes to the node activated by the input and negative for distant nodes (Figure 2B).

A parameter search was initially performed to fit the experimental data with our neural network model. The parameters included: (1) interneuronal distance, (2) variability in input strength, (3) changes in network size, and (4) variability in τ and *T*_*h*_. Parameter sets (1), (2), and (3) involved the effect of inhibition on the neuronal activity. However, they all failed on their own to reproduce the latency distributions in the control condition. So, we did not continue testing them any further in the schizophrenia condition. Only parameter set (4) (changes in τ and *T*_*h*_) reproduced the RT and error rates of the controls. So, we continued our parameter investigation with them in the schizophrenia realm. In each trial run in the left and right SC the integration constants τ of the internal states of each node took values from two different normal distributions with means μ_1_ and μ_2_ and standard deviations σ_1_ and σ_2_, respectively. The model was run for 5000 trials. In each trial we recorded the error prosaccade, antisaccade and corrected antisaccade latency. In the model the error prosaccade reaction time was estimated as the time interval from the onset of the reactive input until the time the activity of the node encoding the reactive input reached a preset threshold plus an additional 30 ms (Figure 2D). The antisaccade reaction time was estimated as the time interval from the onset of the reactive input until the time the activity of the node encoding the planned input reached the threshold plus 30 ms (Figure 2D). The corrected antisaccade reaction time was the time interval from threshold crossing of the error node activity until the threshold crossing of the correct node activity.

**Figure 3:**
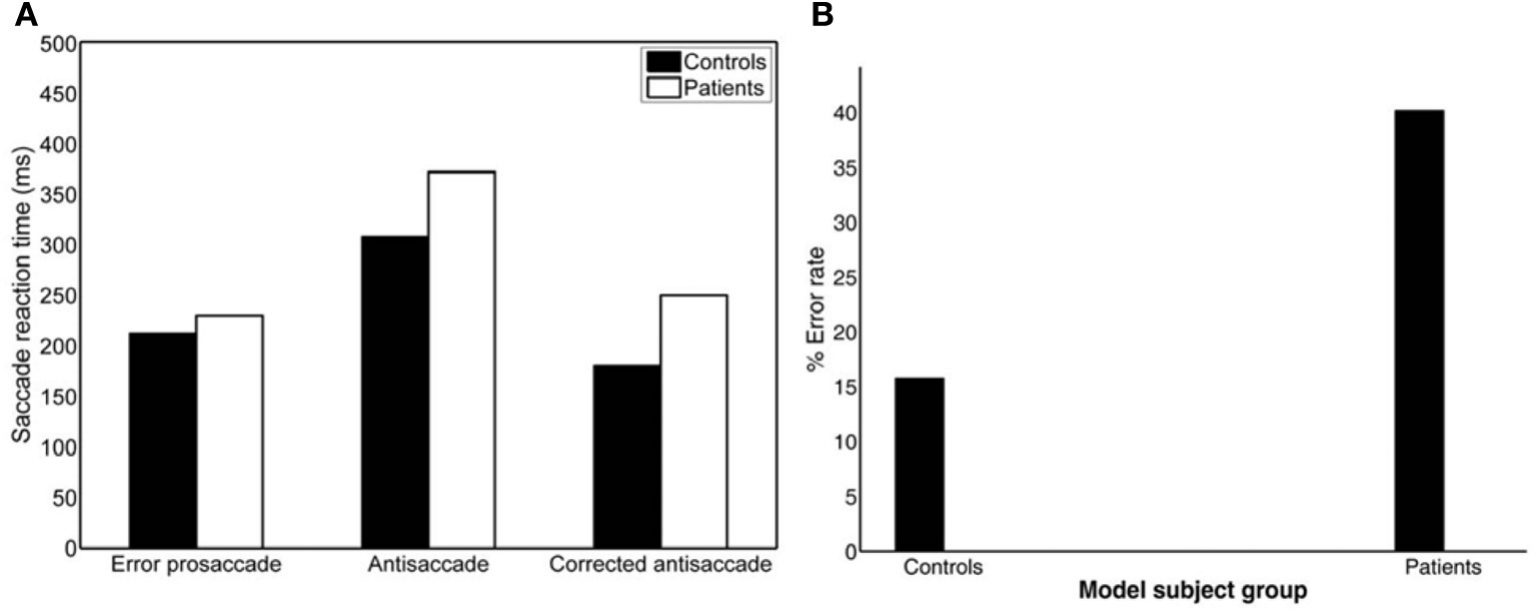
**(A)** Simulated median error prosaccade, antisaccade and corrected antisaccade reaction times (RTs) for controls and patients. **(B)** Simulated percent error rate for controls and patients performing the mirror antisaccade task.

To simulate the error prosaccade, correct antisaccade and corrected antisaccade RTs as well as the error rates and corrected errors we varied the integration constants τ (μ and σ) for both nodes that integrated the reactive (μ_1_ and σ_1_) and planned (μ_2_ and σ_2_) inputs. In the control condition, μ_1_ = 0.01685, σ_1_ = 0.003, μ_2_ = 0.0065, and σ_2_ = 0.0016, whereas in schizophrenia condition μ_1_ = 0.0135, σ_1_ = 0.005, μ_2_ = 0.004, and μ_2_ = 0.002. In both conditions, the threshold value at which as a decision was reached (parameter *T*_*h*_ in Table 3) was constant. The simulated median RTs for the error prosaccades, antisaccades and corrective antisaccades were 212.85, 308.1, and 181.17 ms, respectively for the model controls and 230.32, 372.33, and 250.07 ms, respectively for the model patients. The simulated median RT values are very close to the experimental ones (Figure 3; see also Table 1). The simulated coefficients of variation (CVs) for the error prosaccades, antisaccades and corrected antisaccades were 0.21, 0.17, and 0.44, respectively for the controls and 0.36, 0.28, and 0.5, respectively for the patients. The simulated CV values are very close to the experimental ones (see Table 2).

**Table 3:** **Model parameters**.

**Symbol**	**Value**	**Symbol**	**Value**
*T*_*h*_	0.1791 (0.1791)	σ	2π/10
*C*	0.35	Δ*x*	2π/*N*
*I*_*r*_	1	A	1
*I*_*p*_	1.5	N	100
μ_1_	0.01685 (0.0135)	β	0.5
σ_1_	0.003 (0.005)	θ	0.5
μ_2_	0.0065 (0.004)	μ_*n*_	0
σ_2_	0.0016 (0.002)	σ_*n*_	0.05
*T*	50 ms, unless mentioned otherwise	ntrials	5000

Parameter values in parenthesis represent schizophrenia condition.

**Figure 4:**
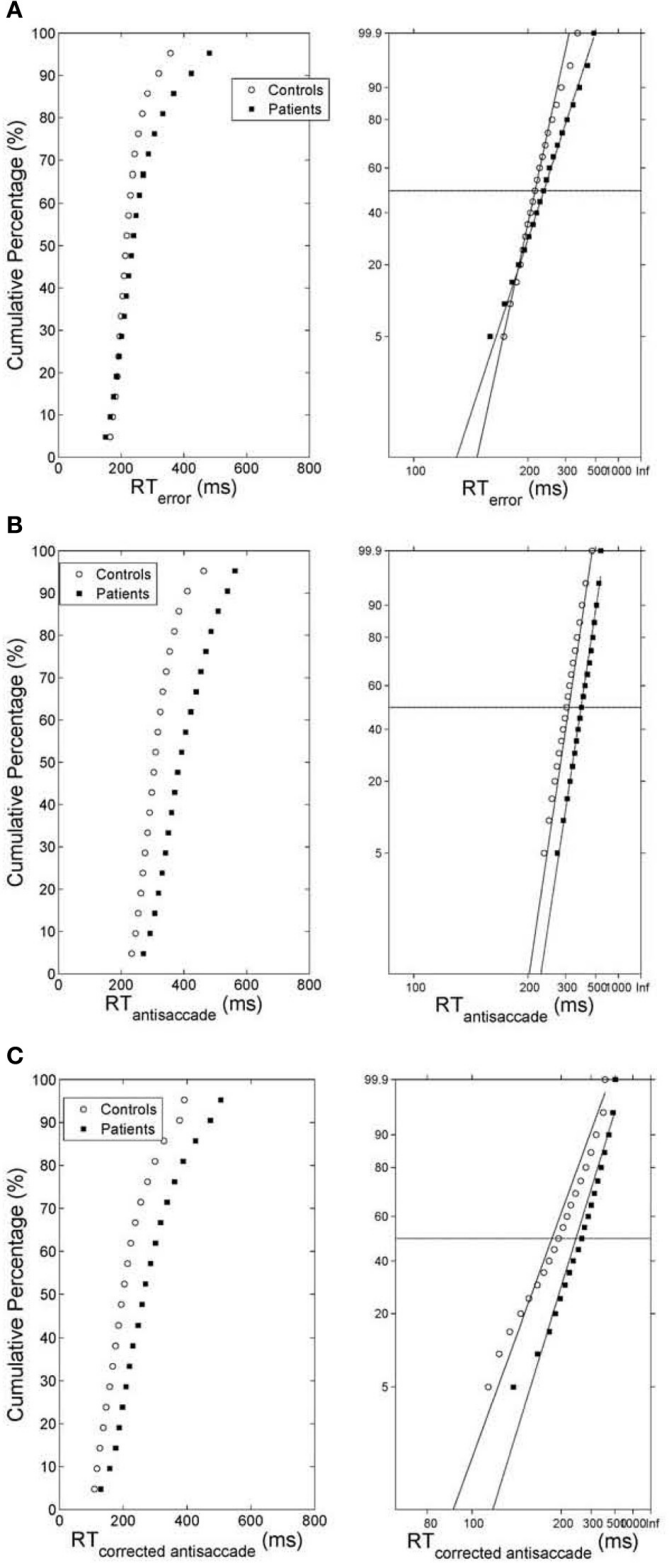
**(A)** (Left) Average cumulative distribution of error prosaccade RT for controls (white empty circles) and patients (black squares). (Right) Reciprobit plots of the average cumulative error prosaccade RT distributions. The x-axis represents 1/RT and it has been reversed so that RTs increase to the right. Instead of 1/RT values the axis is marked with the corresponding RT values. The fitted lines correspond to linear regression on the data of each distribution (controls vs. patients). **(B)** (Left) Average cumulative distribution of antisaccade RT for controls (white empty circles) and patients (black squares). (Right) Reciprobit plots of the average cumulative antisaccade RT distributions. The x-axis represents 1/RT and it has been reversed so that RTs increase to the right. Instead of 1/RT values the axis is marked with the corresponding RT values. The fitted lines correspond to linear regression on the data of each distribution (controls vs. patients). **(C)** (Left) Average cumulative distribution of corrected antisaccade RT for controls (white empty circles) and patients (black squares). (Right) Reciprobit plots of the average cumulative corrected antisaccade RT distributions. The x-axis represents 1/RT and it has been reversed so that RTs increase to the right. Instead of 1/RT values the axis is marked with the corresponding RT values. The fitted lines correspond to linear regression on the data of each distribution (controls vs. patients).

As before we computed the simulated average cumulative RT distributions for error prosaccades, antisaccades and corrected antisaccades for both groups (model controls vs. model patients) by organizing the RT for each subject group from each trial run in ascending order and calculating the percentile values (e.g., the RT for the 5% percentile, the 10% percentile, the 15% percentile, …, the 95% percentile). The percentile values were then averaged across trial runs (5000 trial runs) for each subject group to give average subject group percentile values. We then transformed the average cumulative distribution data of error prosaccade, antisaccade and corrected antisaccade RTs for the simulated controls and simulated patients on a reciprobit plot. A best-fitting regression line was computed for each behavioral category (error prosaccade, antisaccade and corrected antisaccade) in each subject group (controls and patients). The model fit for each behavioral category and for subject group was excellent (correlation coefficient R was 0.99 for error prosaccades and antisaccades and 0.98 for corrected antisaccades in the control subject group and 0.99 for error prosaccades and antisaccades and 0.97 for corrected antisaccades in the patient subject group). The coefficients (slope and intercept) were extracted and fitted to the experimental 1/RT data (see right plots of Figures 4A–C). A comparison of the homogeneity of slopes and intercepts showed that both (controls and patients) fitted error prosaccade lines were statistically different in slope [*t*_(36)_ = 6.01047, *p* = 0.005] and in intercept [*t*_(36)_ = 4.21844, *p* = 0.005]. A similar comparison of the slopes and intercepts were made for the antisaccades and corrected antisaccades for the controls and patients. The fitted antisaccade lines were equal in slope [*t*_(36)_ = 0.209622, *p* = 0.005] and in intercept [*t*_(36)_ = 1.98522, *p* = 0.005]. The fitted corrected antisaccade lines were also found to be equal in slope [*t*_(36)_ = 1.73784, *p* = 0.005] and in intercept [*t*_(36)_ = 0.63875, *p* = 0.005].

### Error rates and errors corrected

The experimental error rate was found to be 27.07% for the controls and 50.26% for the patients (Figure 1C; see also Table 1). In the model an error was considered when the firing activity of the node encoding the reactive input (error prosaccade) crossed a preset threshold level. The model error rate was estimated to be 15.72% for the controls and 40.12% for the patients (see Table 1).

**Figure 5:**
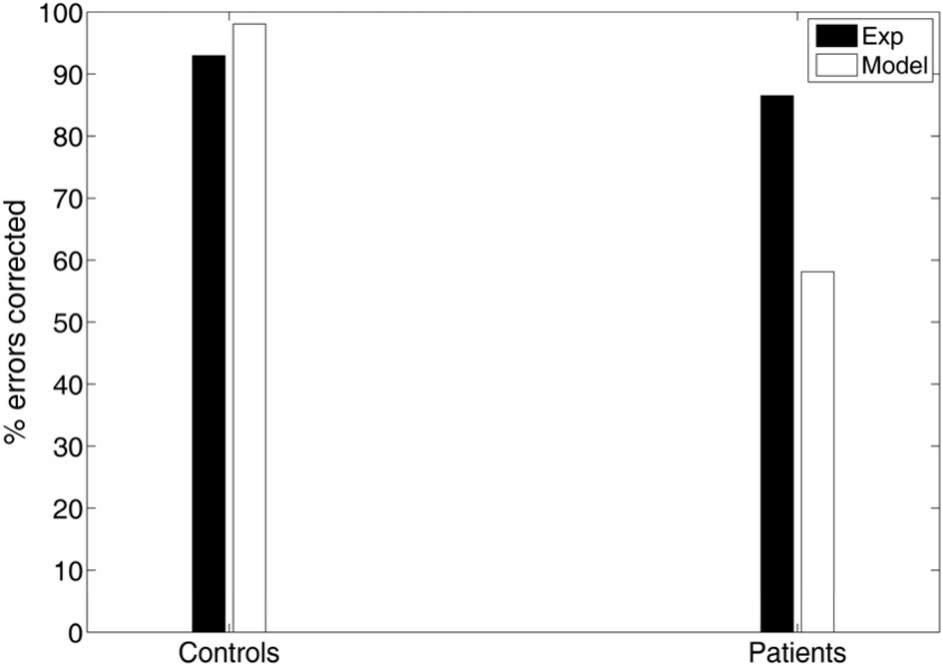
**Experimental and simulated percentage of errors corrected by the controls and patients**.

Another important measure of antisaccade performance is the percentage of errors that are corrected (Hutton and Ettinger, 2006). Everling and Fischer (1998) reported that healthy participants correct the vast majority of errors. However, certain pathological groups fail to correct a significant portion of their errors, suggesting a deficit not only in inhibition, but also in response generation (Guitton et al., 1985; Crawford et al., 2005). In our experimental study controls corrected 93% of their errors, whereas patients have more difficulty in correcting their errors (86.53%; see Figure 5). In the model controls corrected almost all of their errors (98.09%), while patients corrected only 58.13% of their errors (see Figure 5).

### Variability in delay of onset of the planned decision signal input

**Figure 6:**
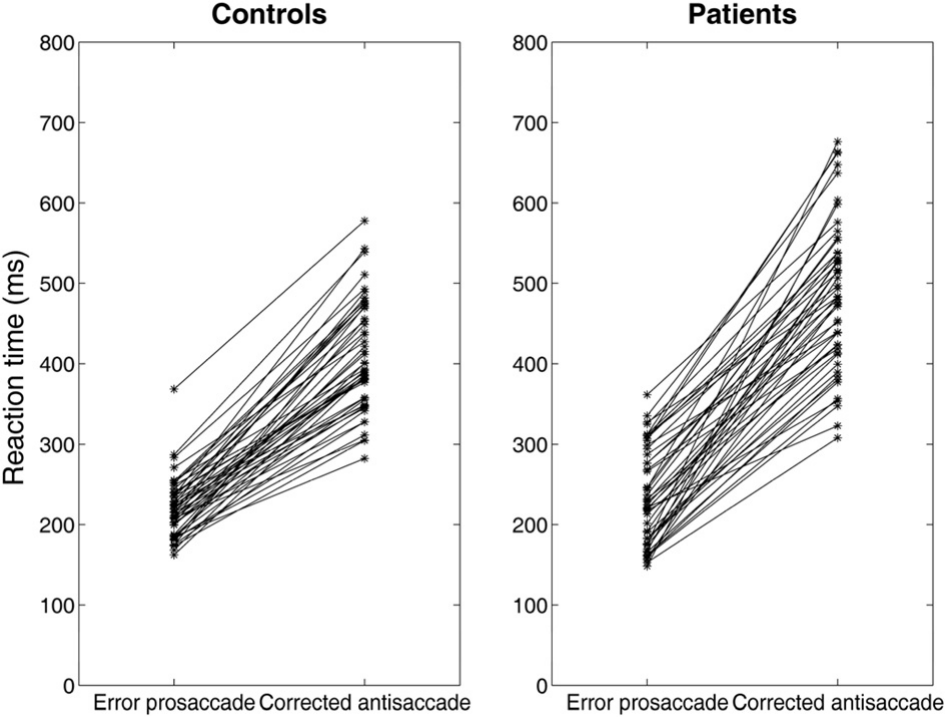
**Median error prosaccade and corrected antisaccade RTs of model controls and model patients performing the mirror antisaccade task**. During each simulation trial the reactive input (error prosaccade) is presented first followed by a fixed time interval *T* = 50 ms by the planned input (antisaccade). Note that the corrected antisaccade latency in this case is the time interval from the threshold crossing of the error node activity till the threshold crossing of the correct node activity plus the error prosaccade latency. The corrected antisaccade RT values are always larger than the error prosaccade ones indicating that the antisaccade eye movement always follows the error prosaccade and not vice versa. Depicted sample RT values were randomly selected from 5000 RTs produced by the model.

Experimental studies of the antisaccade paradigm (Evdokimidis et al., 2002; Smyrnis et al., 2002; Ettinger et al., 2003) have reported that subjects execute only three eye movement behaviors: (1) an error prosaccade toward the peripheral stimulus, or (2) an antisaccade toward the mirror location, or (3) an error prosaccade followed by a corrective antisaccade. At no occasion an antisaccade followed by an error prosaccade is ever observed. Many speculated that this is due to a top–down inhibitory signal arising either from the prefrontal cortex or the basal ganglia that suppresses the error prosaccade when the antisaccade movement have been expressed first (Carpenter, 2000; Munoz and Everling, 2004). In previous modeling studies (Cutsuridis et al., 2007a) and in this study, we showed that such an inhibitory signal may not be necessary and that competition between the error prosaccade and antisaccade encoding neurons can ensure that an error prosaccade movement is never initiated when an antisaccade movement has been executed first (Figure 6). In the model, this behavior takes place only when the antisaccade computation is fast enough and the error prosaccade computation in slow. Then, the antisaccade encoding neuron will cross the threshold first followed by the error prosaccade encoding neuron crossing the threshold. But because, the input simulating the antisaccade is stronger than the one simulating the error prosaccade (*I*_*p*_ = 1.5^*^I_*r*_) and their delay is 50 ms, then as the antisaccade activity increases, then the error prosaccade activity will decrease, thus preventing the error prosaccade from ever being initiated. In Figure 6, the slopes of the straight lines are always positive indicating that corrected antisaccades always follow the error prosaccades, and not vice versa.

**Figure 7:**
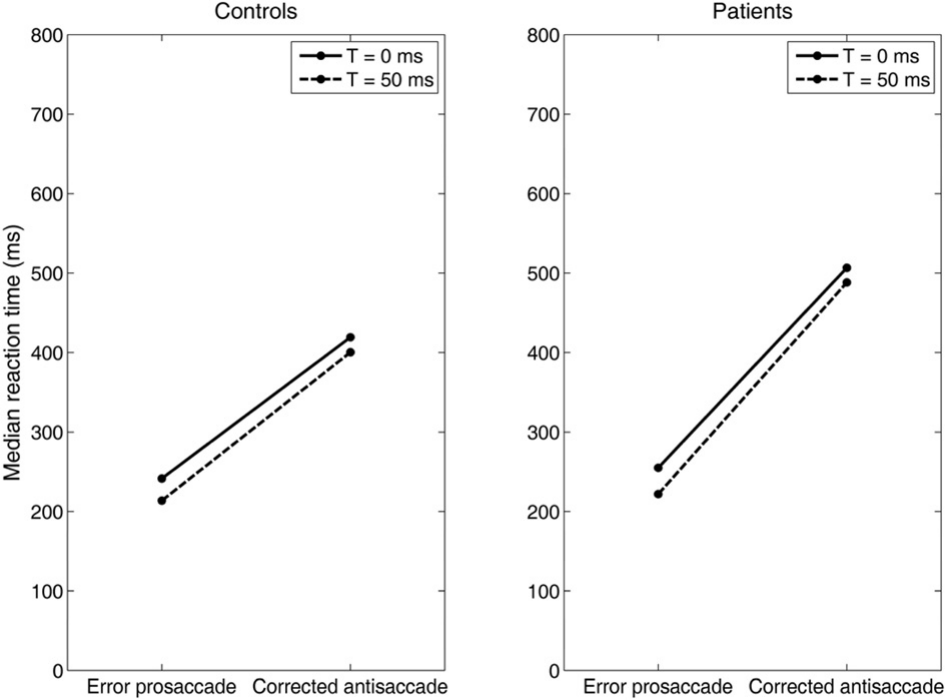
**Median error prosaccade and corrected antisaccade RTs of model controls and model patients performing the mirror antisaccade task**. During each simulation trial the reactive input (error prosaccade) is presented first followed by a variable time interval (*T* = 0 or 50 ms) by the planned input (antisaccade). Note that the RT value of the corrected antisaccade is always larger than the RT value of the error prosaccade for both the controls and patients.

Then, we systematically reduced the onset of the volitional decision signal (antisaccade) (*T* = 30, 10, 0 ms) compared to the onset of the reactive decision signal (error prosaccade) and we observed that the competition between the error prosaccade and antisaccade decision signals and not a third inhibitory signal was sufficient to prevent the error from being initiated when the correct eye movement has been executed first (Figure 7). As before, the slopes of the straight lines are positive indicating that the corrected antisaccades always follow the error prosaccades, and not vice-versa.

## Discussion

Why is the antisaccade performance of subjects with schizophrenia so poor? Why are antisaccade and corrected antisaccade medians greater in subjects with schizophrenia than in controls? Why are latencies more variable and errors greater in patients with schizophrenia? Our model showed that the schizophrenia brains when they performing the antisaccade task are noisier than normal brains. This noise is reflected in the rate of accumulation of information (μ and σ) and not in the baseline activity *S*_0_ (prior probability) or the threshold level *S*_*T*_ (confidence level required before commitment to a particular course of action). As we can see from Table 3 the value of *T*_*h*_ (threshold level *S*_*T*_) is the same in control and schizophrenia conditions meaning that patients with schizophrenia are as confident about their decisions as normal subjects. Studies have shown that the decision threshold level may be set by the basal ganglia (BG) structures (Lo and Wang, 2006). BG dysfunction has been suggested to contribute to the poor antisaccade performance of patients with schizophrenia (Hutton and Ettinger, 2006). Here, we showed that the BG is potentially functioning normally since the decision threshold level (*S*_*T*_) does not change in patients with schizophrenia (see value of *T*_*h*_ in Table 3). However, μ_1_ and μ_2_ (Table 3) are greater in control condition than in schizophrenia condition meaning that error prosaccades, antisaccades and corrected antisaccades are slower for patients with schizophrenia than for normal subjects. Finally, σ_1_ and σ_2_ (Table 3) are smaller in control condition than in schizophrenia condition, which means that error prosaccade, antisaccade and corrected antisaccade latencies are more variable in patients with schizophrenia than in healthy participants. A physiological interpretation of τ and its variability maybe variability of NMDA based rate of evidence integration (Cutsuridis et al., 2007b). Experimental studies have shown that NMDA hypofunction maybe implicated in schizophrenia as well as dopamine and GABAergic inhibition (Lewis, 2012). Dopamine has been shown to modulate the NMDA current (Seamans and Yang, 2004).

Is a third signal, inhibitory in nature, necessary to prevent the error prosaccade from crossing the threshold when the antisaccade reached the threshold first? Such a signal has been speculated to exist (Noorani and Carpenter, 2013), although it has never been experimentally observed. Some have suggested that such an inhibitory signal might originate from the prefrontal cortex or the basal ganglia. A recent study investigating the antisaccade performance of normal participants has suggested that such a STOP process is necessary to suppress the error prosaccade that would otherwise be generated (Noorani and Carpenter, 2013). Their study was successful at simulating accurately the latency distributions of error prosaccades and antisaccades in normal subjects, but not the corrected antisaccades. Our study has provided quantitative evidence that such a third inhibitory STOP process may not be necessary. Competition between the neurons encoding the error prosaccade and antisaccade is sufficient to prevent in some trials the error prosaccade from crossing the threshold when the antisaccade has reached it first. Our model simulated accurately the latency distributions of the error prosaccades, antisaccades and corrected antisaccades in both normal and patients with schizophrenia.

An emergent property of our model is that the corrected antisaccades (median and variance of latency distribution) emerged from the local competition of the error prosaccade and the antisaccade and it was not added to our model as a third process. Noorani and Carpenter (2013) failed to show and simulate corrected antisaccades in their study, although experimental evidence has shown that healthy participants correct the vast majority of errors (generate corrected antisaccades) (Everling and Fischer, 1998). However, certain pathological groups fail to correct a significant portion of their errors, suggesting a deficit not only in inhibition, but also in response generation (Guitton et al., 1985; Crawford et al., 2005). In our experimental study controls corrected 93% of their errors, whereas patients have more difficulty in correcting their errors (86.53%) (Figure 5). In the model controls corrected almost all of their errors (98.09%), while patients corrected only 58.13% of their errors (Figure 5). We believe that the reduced percentage of errors corrected by patients with schizophrenia are due to failure to the sufficiently activate the correct response, which in turn fails to competitively inhibit the erroneous one. This failure is perhaps due to the increased variances of the normal distributions from which the integration constants of the error and correct neuronal activities took values (see Table 3), thus allowing much slower antisaccade computations to take place that never reached the threshold within the 650 ms of simulation time.

Carpenter in his LATER model (1981) proposed if the data are plotted on the reciprobit plot, then the resulting straight line on the reciprobit plot could be used a diagnostic tool to assess the contribution of different factors influencing the experimental results. Changes in a parameter in the LATER model may lead to a specific relation of the straight lines on the reciprobit plot (swiveling, shifting, crossing). In the LATER model, when the lines swiveled by the threshold S_*T*_ (Reddi and Carpenter, 2000), then the mean and variances of the lines were unequal. When the lines were shifted by μ, then the slopes (1/σ) of the lines were equal, but their latency medians were not (Reddi et al., 2003). When the lines crossed, then the slopes were not equal, but their medians were (Nakahara et al., 2006). In our model though we observed that when the lines crossed (error prosaccade; Figure 4A), then the medians are not significantly equal. When the lines shift (antisaccade; Figure 4B), then the medians are significantly different and the coefficients of variations were not significantly different. When the lines swiveled (corrected antisaccades; Figure 4C), then the medians and CVs are significantly different, but the threshold level remained the same (as opposed to the Reddi and Carpenter study 2000). We believe these differences between our model and the LATER model is due to the non-linear nature of our model.

Overall, our model showed in a quantitative way why the antisaccade performance of patients with schizophrenia is so poor, that this performance is not due to a deficit in the top–down inhibitory control of the erroneous response as many speculated, but instead it is a product of a neuronal competition between the erroneous prosaccade and the antisaccade. The model accurately reproduced the error rates, the median antisaccade, median error prosaccade and median corrected antisaccade latencies as well as the antisaccade, error prosaccade and corrected antisaccade distributions of healthy and schizophrenia suffering participants. Our model showed that the increased variability in the antisaccade and corrected antisaccade RT distributions of schizophrenia suffering participants are due to a more noisy accumulation of information (μ and σ) and that their prior expectation (*S*_0_) and confidence level (decision threshold level *S*_*T*_) required before commitment to a particular course of action are not affected by schizophrenia. Further analysis using age as a confounding variable showed that our neural network model's results are independent of any age differences between the groups. A thorough investigation of age as a confounding variable is beyond the scope of this research. More generally, the results presented here illustrate the benefits of tightly integrating psychophysical studies with computational neural modeling, because the two methods complement each other and they may provide together a strong basis for hypothesis generation and theory testing regarding the neural basis of decision making in health and in disease.

### Conflict of interest statement

The authors declare that the research was conducted in the absence of any commercial or financial relationships that could be construed as a potential conflict of interest.
